# Auricular acupuncture for breast cancer-related depression: A protocol for systematic review and meta-analysis

**DOI:** 10.1097/MD.0000000000022870

**Published:** 2020-11-06

**Authors:** Junxiang Gao, Guangling Wu, Enxia Mao, Honglian Zhao

**Affiliations:** a Department of Rehabilitation Medicine of Linyi Central Hospital; b Department of Obstetrics of Linyi Central Hospital, Linyi, Shandong, Province, P. R. China.

**Keywords:** auricular acupuncture, breast cancer-related depression, meta-analysis and systematic review, protocol

## Abstract

**Background:**

Breast cancer is a common disease in galactophore department. Patients with breast cancer are prone to depression, with the incidence of depression ranging from 13% to 23% and as high as 70% in patients with advanced stage. On the one hand, it is related to the physiological characteristics, personality characteristics, and social factors of women themselves. On the other hand, it is related to the common tumors of women, such as breast cancer, examination, and antitumor treatment. Due to the serious side effects of chemotherapy drugs, patients can always feel the presence of cancer. In addition, the mark of radiotherapy and fatigue make the psychological burden continue to have excessive economic burden, and lack of understanding from both social and family, discrimination and patients’ own psychological endurance are all causes of breast cancer factors contributing to depressive symptoms in patients. Auricular acupuncture as a form of acupuncture therapy which is proved to be effective in RCTs and very suitable for patients, has been used in patients who suffer from breast cancer-related depression for a long time, therefore, a systematic review is necessary to provide available evidence for further study.

**Methods:**

The following databases will be searched from their inception to August 2020: Electronic database includes PubMed, Embase, Cochrane Library, Web of Science, Nature, Science online, VIP medicine information, and China National Knowledge Infrastructure. Primary outcomes: Score of depression symptoms. Additional outcomes: The overall effective rate. Data will be extracted by 2 researchers independently, risk of bias of the meta-analysis will be evaluated based on the Cochrane Handbook for Systematic Reviews of Interventions. All data analysis will be conducted by data statistics software Review Manager V.5.3. and Stata V.12.0.

**Results:**

The results of this study will systematically evaluate the effectiveness and safety of auricular acupuncture intervention for people with breast cancer-related depression.

**Conclusion:**

The systematic review of this study will summarize the current published evidence of auricular acupuncture for the treatment of breast cancer-related depression, which can further guide the promotion and application of it.

**Open Science Fra network (OSF) registration number:**

September 11, 2020 osf.io/5b7yw. (https://osf.io/5b7yw)

## 1 Introduction

Breast cancer is a common disease in galactophore department. Patients with breast cancer are prone to depression, with the incidence of depression ranging from 13% to 23% and as high as 70% in patients with advanced stage.^[1]^ On the one hand, it is related to the physiological characteristics, personality characteristics, and social factors of women themselves. On the other hand, it is related to the common tumors of women, such as breast cancer, examination, and antitumor treatment. Due to the serious side effects of chemotherapy drugs, patients can always feel the presence of cancer. In addition, the mark of radiotherapy and fatigue make the psychological burden continue to have excessive economic burden, and lack of understanding from both social and family, discrimination, and patients’ own psychological endurance are all causes of breast cancer factors contributing to depressive symptoms in patients.^[2]^ There is no consensus on the specific treatment in drugs and diagnosis.

Depression is a common affective disorder with high incidence, unclear etiology and pathological mechanism, and complex and diverse symptoms. Throughout development process of modern medicine in the treatment of depression, we found that the early antidepressants because of choline can appear peripheral resistance, cardiovascular and mental abnormal reaction and side effects such as coma. At present, although the safety of drugs to treat depression has improved, but it still have a headache, insomnia, tremor. More importantly, western medicine does not discriminate in the treatment of depression, whose occurrence, development and change is closely related to individual differences.^[3]^ As the therapeutic effect is not satisfactory. Scholars at home and abroad have carried out many studies on the treatment, and the exploration has attracted the attention from many disciplines.

In recent years, acupuncture has been widely used in clinical and experimental studies of breast cancer-related depression and its effectiveness has been fully proved.^[4]^ As a form of acupuncture, the auricular acupuncture has been used to relieve symptoms in patients with breast cancer-related depression, but its effectiveness and safety have not yet reached a definitive conclusion.^[5]^ Therefore, this research intends to adopt the method of system valuation and meta-analysis of the auricular acupuncture for breast cancer-related depression to evaluate the efficacy and safety.

## 2 Methods

### 2.1 Study registration

The protocol of the systematic review has been registered.

Registration: Open Science Fra network (OSF) registration number: September 11, 2020 osf.io/5b7yw (https://osf.io/5b7yw). This systematic review protocol will be conducted and reported strictly according to Preferred Reporting Items for Systematic Reviews and Meta-Analyses^[6]^ statement guidelines, and the important protocol amendments will be documented in the full review.

### 2.2 Inclusion and exclusion criteria for study selection

#### 2.2.1 Inclusion criteria

Inclusion criteria are all randomized controlled trials (RCTs), which main treatment of breast cancer-related depression is auricular acupuncture. The language of the trials to be included only Chinese or English.

#### 2.2.2 Exclusion criteria

Following studies will be excluded:

1. Repeated publications
2. Review of literature and cases
3. Animal studies
4. Incomplete literature
5. Non-randomized controlled trials

### 2.3 Types of participants

The types of subjects included patients diagnosed with breast cancer-related depression, regardless of their degree and possible complications. All patients were treated with auricular acupuncture.

### 2.4 Interventions and controls

Interventions included treatment with auricular acupuncture. The control group only received conventional western medicine treatment. The routine treatment of each RCT may not be identical, but the use of auricular acupuncture is the only difference between intervention and control.

### 2.5 Types of outcome measures

#### 2.5.1 Main outcomes

1. Score of depression symptoms.

#### 2.5.2 Additional outcomes

1. The overall effective rate;

### 2.6 Search methods

#### 2.6.1 Search resources

**Figure 1:**
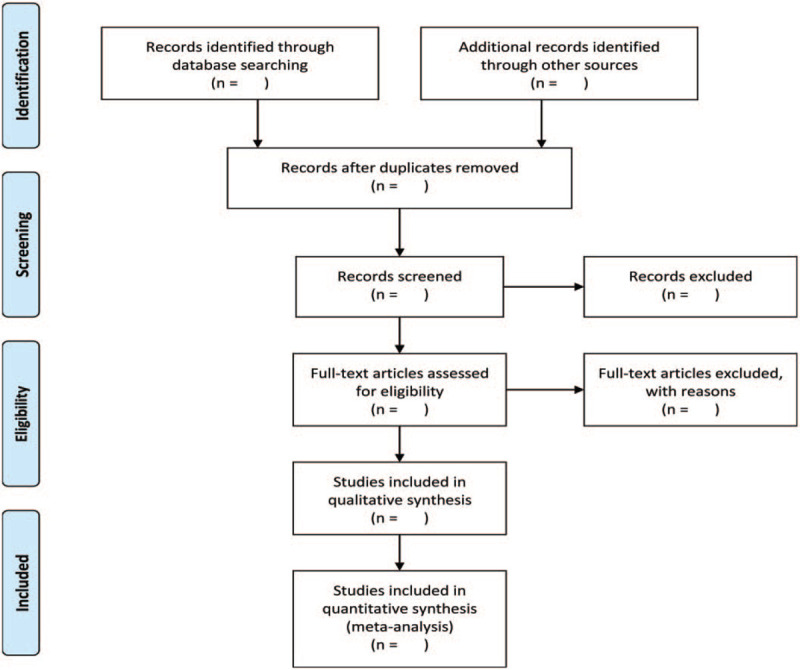
The research flowchart. This figure shows the Identification, Screening, Eligibility and Included when we searching articles.

We will search the following electronic databases from their inception to August 2020: Electronic database includes PubMed, Embase, Cochrane Library, Chinese Biomedical Database, VIP medicine information, and China National Knowledge Infrastructure (Fig. 1). The research flowchart.

#### 2.6.2 Search strategies

The following MeSH terms and their combinations will be searched:

(1) auricular acupuncture;
(2) RCT OR RCTs;
(3) breast cancer-related depression (Table 1). The research strategy.

**Table 1:** Search strategy sample of PubMed.

Number	Searchs
#1	Breast cancer-related depression (MeSh)
#2	Breast cancer depression (ti,ab)
#3	Depression after breast cancer (ti,ab)
#4	Depression associated with breast cancer (ti,ab)
#5	or#1–4
#6	Medicine, Chinese Traditional(MeSh)
#7	Traditional Chinese medicine(ti,ab)
#8	TCM(ti,ab)
#9	or# 6–8
#10	auricular acupuncture (MeSh)
#11	auricular acupuncture (ti,ab)
#12	auricular needle (ti,ab)
#13	ear acupuncture (ti,ab)
#14	auricular point sticking (ti,ab)
#15	auricular point (ti,ab)
#16	or#10-15
#17	Randomized Controlled Trial (MeSh)
#18	Randomized Controlled Trial(ti,ab)
#19	RCT(ti,ab)
#20	or#16-19
#21	#5 and #9 and #16 and #20

### 2.7 Data collection and analysis

#### 2.7.1 Studies selection

There will be 2 researchers (JG and GW) carry out the selection of research literature independently using Note-Express software. We will first make the preliminary selection by screening titles and abstracts. Second, we will download full text of the relevant studies for further selection according to the inclusion criteria. If there is any different opinion, 2 researchers will discuss and reach an agreement. If a consensus could not be reached, there will be a third researcher (EM) who make the final decision. The details of selection process will be displayed in the Preferred Reporting Items for Systematic Reviews and Meta-Analyses flow chart.

#### 2.7.2 Data extraction

Two researchers (JG and GW) will read all the included text in full, and independently extract the following information:

(1) general information, including trial name and registration information;
(2) trial characteristic, including trial design, location, setting, and inclusion/exclusion criteria;
(3) the characteristics of the participants, including age, race/ethnicity, course of illness, and so on;
(4) details of intervention, including acupoints, time of intervention, course of treatment, time of single treatment, and so on
(5) details of comparison interventions;
(6) outcomes as described under type of outcome measure section.

If we could not reach an agreement, a third researcher (EM) would make the final decision. One researcher (HZ) would contact the corresponding author by telephone or e-mail for more information when the reported data were insufficient or ambiguous.

#### 2.7.3 Assessment of risk of bias

All the included studies will be evaluated based on the guidelines of Cochrane Handbook for Systematic Reviews of Interventions.^[7]^ The quality of each trial will categorized into “low,” “unclear,” or “high” risk of bias according to the following items: adequacy of generation of the allocation sequence, allocation concealment, blinding of participants and personal, blinding of outcome assessors, incomplete outcome data, selected reporting the results and other sources of bias (such as comparable baseline characteristic, inclusion and exclusion criteria).

#### 2.7.4 Assessment of reporting biases

Reporting biases and small-study effects will be detected by funnel plot and Egger test if there are 10 more studies included in this Meta-analysis. For Egger test, *P*-value of <0.10 was considered to indicate the exist of reporting biases and small study effects.

#### 2.7.5 Data analysis

We used Revman 5.3 software provided by the Cochrane collaboration to analyze the data. Binary outcomes will be summarized using risk ratio with 95% confidence interval for relative effect. Continuous outcomes will be summarized by using weighted mean difference with 95% confidence interval. We will use random-effect model for meta-analysis in this review according to research recommendations.^[8]^

Statistical heterogeneity will be assessed by *X*^2^ and *I*^2^ statistical tests. Where *P*-value ≥ .1 and *I*^2^ ≤ 50%, there is no obvious statistical heterogeneity among the studies. On the contrary, where *P*-value < .1or *I*^2^ > 50% indicates a considerable heterogeneity. Meta-analysis will be performed when the statistical heterogeneity is acceptable (*P*-value ≥ .1 and *I*^2^ ≤ 50%), otherwise, subgroup analysis will be applied to explore the influence of potential factors on the outcome measures. We will conduct sensitivity analyses by omitting studies one by one in order to probe the impact of an individual study. If a meta-analysis cannot be performed, we will conduct descriptive analysis instead.

#### 2.7.6 Patient and public involvement

This is a meta-analysis study based on previously published data, so patient and public involvement will not be included in this study.

#### 2.7.7 Ethics and dissemination

Ethical approval will not be required as this is a protocol for systematic review and meta-analysis. The findings of this study will be disseminated to a peer-reviewed journal and presented at a relevant conference.

#### 2.7.8 Evidence assessed

The quality of evidence for this study will be assessed by “Grades of Recommendations Assessment, Development and Evaluation (GRADE)” standard established by the World Health Organization and international organizations.^[9]^ To achieve transparency and simplification, the quality of evidence is divided into 4 levels in GRADE system: high, medium, low, and very low. We will employ GRADE profiler 3.2 for analysis.^[10]^

## 3 Discussion

So far, breast cancer has become the most common malignant tumor in women worldwide, and it is also the main cause of female cancer death.^[11]^ A recent study of the incidence, survival, and mortality rates of breast cancer in women worldwide based on publicly published global data^[12]^ indicates that in 2008, approximately 1.4 million women were diagnosed with breast cancer worldwide, and nearly all women died from the disease. Studies have shown that the psychological disorders of cancer patients are usually manifested as varying degrees of depression.^[13,14]^

Western medicine mainly users antidepressant therapy, which has certain effect, but it needs to be taken for a long time, and the adverse reactions are obvious. Cancer patients, especially advanced patients, are often accompanied by multifunctional organ damage, so the treatment plan has to be further optimized. Traditional Chinese medicine therapy can regulate the functions and functions of the viscera in a benign way, which has attracted a lot of attention from researchers.^[15]^

Therefore, as a traditional Chinese medicine, auricular acupuncture has its own characteristics unique advantages, which can alleviate depression symptoms. The theory of Traditional Chinese medicine shows that Auricular acupuncture can adjust the viscera qi and blood, Yin and Yang of the human body, and auricular points depend on the human body. The theory that all parts of the body are holographic and reflected in the auricle can cure Zang-Fu Diseases through the stimulation of the seeds.

Auricular acupuncture is a painless treatment method which does not directly stimulate the nerve endings of pain sensation, so it does not produce pain. According to research on clinical treatment, it very effective to treat breast cancer-related depression by stimulating auricular points. The patients compliance greatly improved because of this advantage. In addition, compared with common acupuncture, auricular acupuncture has thin and short body which can get more security. Long-term embedding of the intradermal needle into the subcutaneous myofascial layer can maximize the electrochemical effect of acupuncture. Long-term stimulation can regulate the plant nerve function, promote blood circulation, and achieve better curative effect.^[16,17]^

This systematic review will evaluate published RCTs evidence for the effectiveness and safety of auricular acupuncture for breast cancer-related depression. This study has several strengths, it may assist clinicians and patient treatment for breast cancer-related depression with guidelines. Clinical research will be conducted based on this systematic review protocol. In general, this review will be the first to evaluate the effects of auricular acupuncture on breast cancer-related depression outcomes. On this basis, a better treatment method can be established to provide a reliable basis for its wide application.

## Author contributions

**Conceptualization:** Guangling Wu, Junxiang Gao

**Data curation:** Guangling Wu, Honglian Zhao, Junxiang Gao.

**Formal analysis:** Enxia Mao, Guangling Wu, Honglian Zhao.

**Methodology:** Enxia Mao, Guangling Wu, Junxiang Gao.

**Project administration:** Enxia Mao.

**Resources:** Enxia Mao, Guangling Wu, Junxiang Gao.

**Software:** Guangling Wu, Junxiang Gao.

**Supervision:** Enxia Mao.

**Writing – original draft:** Junxiang Gao.

**Writing – review & editing:** Junxiang Gao.
